# Emergent Neuroimaging Findings for Written Expression in Children: A Scoping Review

**DOI:** 10.3390/brainsci12030406

**Published:** 2022-03-18

**Authors:** Lara-Jeane C. Costa, Sarah V. Spencer, Stephen R. Hooper

**Affiliations:** Department of Health Sciences, School of Medicine, University of North Carolina-Chapel Hill, Chapel Hill, NC 27516, USA; lara-jeane_costa@med.unc.edu (L.-J.C.C.); sarah.vanselous@gmail.com (S.V.S.)

**Keywords:** written expression, brain imaging, neuropsychology, children, neuroimaging and writing

## Abstract

Background: There is currently a dearth of research on the neural framework of writing tasks in children, as measured by neuroimaging techniques. Objective: This paper provides an overview of the current literature examining the neurological underpinnings of written expression in children. Design: Using a scoping review approach, with thorough searches of key databases, this paper presents the available literature comprising 13 different studies using both structural and functional neuroimaging techniques with the 0–18 English speaking population. Results: Studies largely presented small sample sizes, with most studies utilizing elementary or middle school-aged children. Emergent findings revealed a complex network of neural contributions to the writing process in children. There were associations between the left fusiform gyrus and orthographic coding (i.e., handwriting), and spelling and written composition measures were significantly correlated with activity in the left posterior cingulate, left precuneus, and right precuneus regions. Additionally, results revealed that good versus poor writers manifested differential brain activation patterns during many tasks associated with written expression, with good writers performing more efficiently than poor writers with respect to brain regions activated during a writing task across handwriting, spelling, and idea generation. Conclusions: The findings from this scoping review lay the foundation for future studies examining the interface between writing skills in children and underlying neural pathways that support the various components of the writing process. It will be important for future research to examine the neurological bases of the various components of written expression in children and adolescents.

## 1. Introduction

Prior to the 20th century, writing was considered no more than a final product of motor output; conversely, during the second half of the 20th century, researchers and educators began to view writing as a complex cognitive process [1]. Much of the research since that time has explored the area of written expression from a neurocognitive perspective. It is evident now that writing is an extremely nuanced and demanding set of skills that involves many cognitive processes (e.g., planning, revising, spelling, transcription, text-generation) to produce readable text. Additionally, writing is a self-sustained activity that requires a great deal of cognitive effort to orchestrate the numerous processes, knowledge, and skills involved in composing tasks [2]. Applying neuroimaging techniques to writing research can help to elucidate these purported connections between specific writing components and their underlying neurological systems and to provide another avenue to examine response to interventions, improving writing instruction. For this reason, a scoping review was completed to gain a better understanding of what has been learned about writing through neuroimaging, and identify areas of further research.

### Objective of the Scoping Review

To date, empirical studies have utilized brain imaging techniques to examine the neurological underpinnings for a variety reading and mathematics components in children; however, there has not been a compilation of studies that have examined the use of brain imaging strategies for written expression. One reason for this is that the process of writing itself is complicated and, pragmatically, there is significant motor movement involved in writing that can interfere with the quality of the brain image obtained secondary to movement artifact. Nonetheless, there is a slowly growing literature that uses neuroimaging techniques to examine written expression in children. As such, the objective of this scoping review is to provide an analysis of the emergent empirical research in neuroimaging of children and written expression. In addition to reviewing the available findings, we will discuss how these emergent findings have the potential to inform both the theory and practice of writing instruction and response to intervention in children. Finally, we discuss future research innovations and challenges in the intersection between neuroimaging and written expression, with a particular focus on the application of these findings to classrooms and other instructional settings. A scoping review was utilized as the primary method for gathering studies given the relative dearth of studies available to date, but also to: (1) identify the available evidence in the neuroimaging of writing in children, (2) clarify key findings that have emerged from this literature, (3) explore the various neuroimaging techniques employed in these investigations to date, and (4), in line with the overall goal of a scoping review, identify gaps in the literature and lay the foundation for future studies [3].

## 2. Methods

Utilizing the Preferred Reporting Items for Systematic Reviews and Meta-Analysis extension for Scoping Reviews (PRISMA-ScR) [4] structure for conducting scoping reviews as a guide, studies were included if participants were between 0 and 18 years of age, researchers used neuroimaging techniques to collect observational data (not instructional or experimental), and participants completed a task related to written expression either in or outside the scanner. We excluded studies that examined participants with traumatic brain injuries (e.g., strokes), studies not written in English or offering no English translation, studies involving writer’s cramp, and studies with non-native English speakers. The reason for the last exclusion criteria was that a previous study conducted by Borkowski et al. [5] highlighted that “the specific nature of the language and the type of the task are important variables underlying the clinical picture of spelling disorders as well as their psychological and neuropsychological mechanisms, indicating that variances among languages may affect the regions of the brain associated with the many processes involved in written expression.”

**Figure 1:**
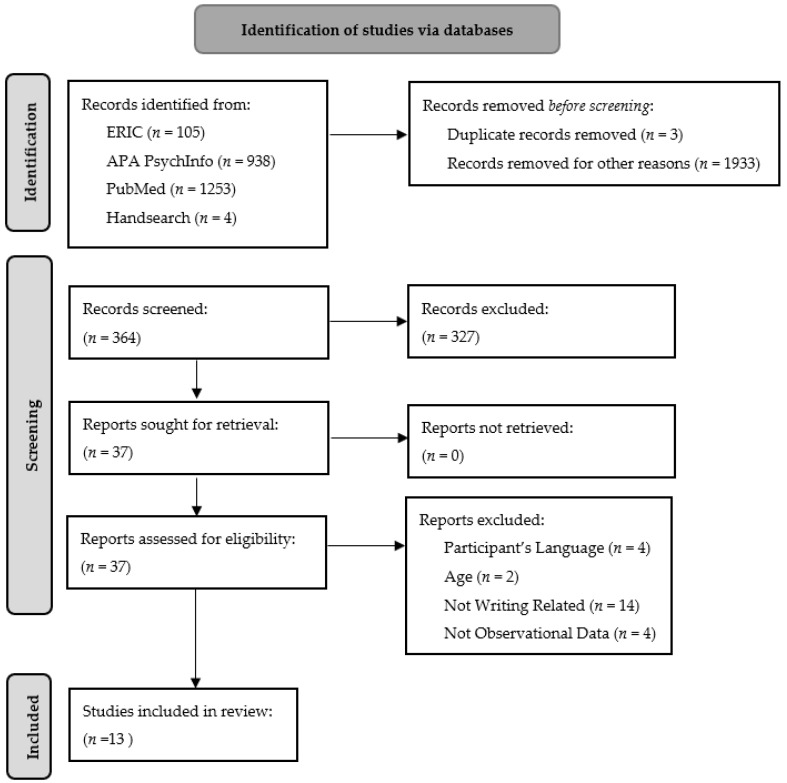
Scoping review flow diagram.

A systematic search was conducted in February of 2022 via the databases ERIC, APA Psychinfo, and PubMed using the same search string in each database: (BOLD OR fMRI OR “functional magnetic resonance”) AND (handwriting OR “letter form” OR spell* OR essay OR “idea generation”) with limits based on our exclusion criteria (e.g., age, language, species) and with no date range specified. This resulted in 2300 hits across all included databases. The search string was developed by the principal investigator and peer-reviewed by all authors. Gray literature was not searched. Duplicates were removed, and two research associates double screened all identified articles by title and abstract for inclusion and exclusion criteria. Any disagreements were resolved through discussion with other reviewers. After review, nine articles were revealed to match inclusion and exclusion criteria. Hand searching based on the introductions and conclusions of the articles revealed an additional 4 articles that did not come up in the formal search. This systematic search can be seen in Figure 1. Three reviewers read each included article for understanding and discussed findings. A data-chart form was developed by two reviewers in a collaborative process. For each article, one reviewer contributed article information to the data-chart, which was reviewed by at least one other reviewer for clarity and accuracy. Data was extracted on the population age (e.g., years or school grade), sample size, the neuroimaging methodology used (e.g., fMRI, MRI), the specific writing task participants were asked to complete, and the related brain regions identified through neuroimaging. The result is shown in Table 1, which includes all 13 articles that matched inclusion criteria.

Studies were grouped by the area of writing that was primarily examined (i.e., handwriting, spelling, idea generation/planning), and summaries were created of the methodologies and broad findings in our results. Particular attention was given to the types of neuroimaging techniques used and specific brain regions identified by the studies, to be able to synthesize overlapping findings between studies and obtain our objectives.

## 3. Results

Results of the scoping review yielded 13 studies that focused on the use of neuroimaging techniques to examine the neurological underpinnings of key writing components in children. These key components included handwriting (four studies), spelling (five studies), and idea generation/planning (two studies). Two additional studies addressed multiple writing components and were included under handwriting [9,10]. To date, there are no neuroimaging studies in children examining text generation during the imaging procedure, although along with idea generation a number of studies have examined planning as a precursor to text generation. While several studies used structural Magnetic Resonance Imaging (MRI) in their investigations (i.e., diffusion tensor imaging), all the available studies employed functional Magnetic Resonance Imaging (fMRI) and/or Diffusion Tensor Imaging (DTI) to examine a specific writing component. A description of these studies can be found in Table 1 and are explained in further detail below.

### 3.1. Handwriting

Richards et al. [6] examined the differences in letter production (i.e., familiar and pseudo-letters) between good and poor 11-year-old writers using fMRI contrasts. Writing ability was determined using behavioral measures of handwriting and text generation. The participants were trained on the handwriting task prior to entering the fMRI scanner. In order to keep motor requirements constant between familiar and pseudo-letters (i.e., newly taught letter form), students were instructed to use the “ball and stick” method of constructing letter forms; for instance, the real letter “ɑ” was written as a ball with a stick to the right, and a pseudo-letter as a ball with a stick below.

When contrast data between the practiced letter form and pseudo-letter form were analyzed based on a group map, the researchers found significant BOLD activation for both groups of writers in many similar areas of the brain (i.e., left calcarine, right lingual, right cerebellum regions 4, 5, 6, and 9, left and right cerebellum region 8, and vermis regions 6, 7, 8, and 9) indicating neurological similarity in handwriting regardless of skill level. The good writers, however, only activated an additional 5 areas of the left side of the brain (i.e., precentral, postcentral, inferior parietal, cerebellum 7b, and cerebellum 9), while the poor writers activated an additional 13 areas on both sides of the brain (i.e., left posterior cingulum, right calcarine, left and right cuneus, left lingual, left superior occipital, left middle occipital, left fusiform, left superior parietal, left and right precuneus, left and right cerebellum crus 1, right cerebellum crus 2, left cerebellum 6, and vermis 4 and 5). Richards et al. [6] suggested that poor writers have less neural efficiency than good writers when writing a newly taught letterform, since poor writers activated more brain regions and different sets of brain regions than good writers.

In the same study, Richards et al. [6] examined BOLD activation patterns based on individual brain activation maps. Their analysis revealed that good writers activated significantly more in the left fusiform region, which contains the visual word form area (VWFA), than poor writers. This was significantly correlated with behavioral measures (i.e., automatic letter writing and expressive orthographic coding). The authors concluded that the results validated the connections between the left fusiform gyrus and orthographic coding for 11-year-olds.

In another study, Giminez et al. [7] examined neurological associations to handwriting by examining brain activity of 46 5- and 6-year-old writers whose handwriting ability had been ranked. In the scanner, the children completed three tasks including a phonological processing task in which participants had to determine whether the first sound of the names of two pictures of common objects matched, and two other tasks that required judgment of visual symbols without the sound-matching aspect. Findings showed stronger activity in the right inferior frontal gyrus during the sound-matching task was associated with poorer handwriting quality. The potential implication here is that the inferior frontal gyrus may be a critical component for the development of the motor demands to engage in handwriting, and that the development of both handwriting skills and phonological processing capabilities may be interdependent.

Richards et al. [8] used both structural and functional neuroimaging to examine brain differences in 4th through 9th graders (9–15-year-olds). Participants were placed into three different groups: Typicals, Dyslexia, and Dysgraphia. On structural connectivity, the Dysgraphia Group showed less white matter integrity in the bilateral anterior thalamic radiation, left cingulate, and forceps minor than either of the other two groups. They also manifested less integrity in the left cortical spinal tract when compared to Typicals. The Dysgraphia Group also showed higher radial diffusivity in seven white mater tracts in the left hemisphere than the Dyslexia Group. No differences were observed between the Typical versus Dysgraphia Group on the resting condition or, surprisingly, on the alphabet writing task.

Using three fMRI writing tasks including writing the next letter in the alphabet, adding the missing letter in word spelling, and planning for composing, Richards et al. [9] examined the brain connectivity of the cingulo-opercular network in children who were in grades 4–9 (mean age = 11 years, 10 months). Specifically, these investigators found that significant fMRI connectivity with this network was uncovered in left cingulate gyrus during the fMRI alphabet writing task. These findings highlight the importance of this network in the regulation of written output.

In a small sample of children (*N* = 13) with dysgraphia in later elementary and middle school, Richards et al. [10] used fMRI to examine the impact of ADHD during four writing tasks. Two of the tasks were cognitively based (i.e., mind wandering, planning to compose) and two were transcription based (i.e., handwriting, spelling). The presence of ADHD was significantly related to the degree of brain connectivity across all three of the four writing tasks. Specifically, for the cognitively based writing task of mind wandering, after correction for multiple comparisons, significant correlations were noted for the left occipital temporal with fusiform 2, and left supramarginal with fusiform 2. For the planning task, no significant correlations were observed after correction for multiple comparisons. For the transcription writing tasks, significant correlations were noted between the left supramarginal region and Broca’s area, for both alphabet writing and spelling tasks. In general, children with dysgraphia and ADHD showed significantly more functional connectivity across all tasks during fMRI, suggesting less efficiency in brain functioning during those types of writing tasks. This study further implicates the complexity of neuroimaging during writing and shows the impact of comorbid conditions on brain activity during the writing process. How other comorbid conditions (e.g., anxiety) might affect the brain connectivity remains to be determined.

As a derivative of handwriting, Richards et al. [11] investigated BOLD activation during finger succession and finger repetition tasks. The authors hypothesized that (1) BOLD activation would differ between good and poor writers on the two tasks and (2) activation on the fMRI contrast for sequential versus nonsequential finger movements would be predicted by behavioral measures (i.e., finger sequencing, handwriting, and spelling). The sample included 12 good writers and 8 poor writers. These participants were recruited from a 5-year longitudinal study during which writing ability was assessed with the same behavioral measures as in Richards et al. [6]. Data were collected for this study between 5th and 6th grade. While in the scanner, participants acted within two conditions: an on-task in which participants tapped fingers in succession, and an off-task in which participants tapped the same index finger repeatedly. The results suggested that several key brain regions previously supposed to be important to written expression (i.e., left superior parietal, right inferior frontal orbital, right and left inferior temporal areas) were activated in good, but not poor, writers. As well, BOLD activation in these areas was significantly correlated with handwriting, spelling, and composing derived outside the scanner.

### 3.2. Spelling

Bitan et al. [12] used fMRI BOLD activation to investigate orthographic language (visual spelling) with 38 children ages 9–15-years-old. While in the scanner, participants were shown 24-word pairs, one word at a time, to determine if the paired words had the same rime (letters that follow the initial phonological unit of a word) to assess spelling. The task was similar during the control condition, except instead of words the participants were shown symbol pairs. Even though results showed greater activation during the spelling task than the symbol task in the perisylvian language areas and the left infero-temporal region; results also showed that participants relied heavily on nonlinguistic processes while deciding between the word pairs.

Using the same spelling task as in the Bitan et al. study, Booth et al. [13] examined developmental increases in effective connectivity to left hemisphere brain regions with 48 children ages 9–15-years-old. Their results showed developmental increases in the effective connectivity from calcarine to the superior temporal gyrus. They suggested that the modulatory effects are not from brain development within each region, but a result of development of the pathways and their use during the task.

Berninger et al. [14] used behavioral and neuroimaging data to supplement each other in a study involving children in grades 4–9. Participants were given a battery of assessments to categorize them into dyslexia, dysgraphia, and oral written expression disability diagnostic groups. Following this categorization, participants were asked to complete a word-specific spelling judgment task during the fMRI scan. Findings revealed that students with different diagnoses had different brain patterns in terms of which parts of the brain were activated during the task as well as how many regions. This evidence confirms that these disorders, while similar, have significantly different neural manifestations, which should be further explored to determine differences in their neurodevelopmental ontogeny, as well as the potential instructional needs of these students. In a related study using a similar sample, Richards et al. [8] found that the Dysgraphia Group evidenced DTI-fMRI functional connectivity correlations between the left supramarginal gyrus and the left cingulum the anterior cingulate gyrus, and between the left inferior frontal regions and the superior parietal lobule, superior frontal gyrus, and the precuneus cortex on the blank spelling task.

Richards et al. [15] investigated BOLD activation differences between 11-year-old good and poor spellers during access to working memory. Spelling skills were determined by spelling and composing components of the Wechsler Individual Achievement Test-II (WIAT-II) (i.e., Verbal Comprehension Index). The study explored two kinds of orthographic representations: (1) temporary, characterized by partial, incomplete representations formed when encountering an unfamiliar word form; and (2) long-term, characterized by durable, lasting representations formed through repetition. Specifically, they examined how good and poor spellers differ in accessing temporary and long-term orthographic representations in working memory as they performed two different spelling tasks during an fMRI scan. The spelling tasks required participants to make judgments about (1) pseudowords while they were displayed versus from memory, and (2) pairs of words that were either both correctly spelled real words or one correctly spelled word and one word that could be pronounced as a real English word but is not. The results suggested significant correlations between WIAT-II composition and brain activation in three areas: the left posterior cingulate gyrus, left precuneus, and right precuneus. Good and poor spellers differed significantly in the rate of pseudoword reading; the authors also found positive correlations between pseudoword reading rate and good spellers’ brain activation, and negative correlations between pseudoword reading rate and poor spellers’ activation. Taken together, these findings point to poor spellers showing an over-activation of the left superior frontal gyrus (i.e., access of long-term orthographic representations). At the same time, good spellers showed more activation in the right superior frontal gyrus, which may suggest the neurological underpinnings for higher-order executive function of translation and the related automaticity of orthographic representation in spelling—efficiently turning cognitive representations (e.g., concepts underlying vocabulary words, ideas) into written words.

Finally, Richards et al. [16] used fMRI techniques to investigate the engagement of working memory in children with and without a spelling disability in order to examine whether good and poor spellers engage working memory differently. The participants’ ages ranged from 10 to 12 years old and included 10 good spellers and 20 children with an identified spelling disability. While in the scanner, there were two working memory tasks asked of the participants: a 0-back control condition and a 2-back condition. The stimuli presented were colored pictures of sea creatures. The 0-back control task asked the children to judge if the picture presented was the target picture (i.e., whale), and the 2-back condition required the participants to judge if the picture matched the one presented two trials earlier. When brain activation maps were analyzed, important differences were recognized in the distribution and degree of neural activity during the tasks, suggesting that working memory was not concentrated in one neural system but, rather, spread spatially and temporally. The poor spellers activated significantly more brain clusters than the good spellers, particularly in the frontal and cingulate regions. This distribution of neural effort across more brain regions suggested that the working memory of poor spellers is inefficient and does not facilitate the management of the processes involved with spelling.

### 3.3. Idea Generation

While no neuroimaging studies have yet been conducted to examine text generation, Berninger et al. [17] investigated idea generation in written expression using both behavioral and fMRI methods. This was a 5-year longitudinal study with the sample comprising beginning first grade students. Of the 128 enrollees, 20 students were selected for the fMRI subsample. In the behavioral task, students were asked to verbally generate ideas about two topics (i.e., computers and robots). These responses were transcribed, examined, and categorized into 24 groups that reflected the nature of the idea generated. The behavioral data were used to predict BOLD activation. For the imaging portion, participants generated ideas while in the fMRI scanner. There were two tasks asked of the participants in the scanner: an off-task and an on-task. During the off-task the participants were asked to just rest and for the on-task they were prompted: “Think about what you learned this summer that you have never learned in school. In a little while, you will leave the scanner and write about this topic.” BOLD activation results revealed that good writers showed more activation than poor writers in brain regions previously found to be associated with access to cognitive concepts and higher order cognition (left and right superior frontal gyri), language and executive functions related to language (left inferior frontal gyrus), working memory (right middle frontal orbital gyrus), and coordination (right cerebellum). In contrast, poor writers activated more than good writers in one region in the left hemisphere, which is associated with working memory (left middle frontal gyrus). Their results suggested that poor writers are inefficient in engaging working memory during idea generation.

Richards et al. [8] reported on a planning to write task while in the scanner for their 9–15-year-old sample. For their Dysgraphia Group they found greater functional connectivity during planning in the left occipital temporal, left inferior frontal gyrus, and left precuneus than the Typical Group. This over-connectedness suggests less efficiency during the planning process than what might be seen in typically developing writers and lays the neural foundation for poor text production.

In a related study, Wallis et al. [18] examined differential patterns of fMRI connectivity in students with and without problems in transcription in grades 4 through 9. The students with problems included those with dyslexia or dysgraphia. During fMRI, all participants complete writing tasks that included a resting state, alphabet writing, spelling writing, and planning. For the dysgraphia group, fMRI connectivity for three of the four tasks was significantly related to one or more of the composition outcomes created outside the scanner. Handwriting was the only exception. The significant correlations for spelling were observed from left precuneus with the fusiform gyrus and amygdala, and from left inferior frontal gyrus with Broca’s area and fusiform gyrus. For planning, the connectivity was observed with left precuneus with cingulate, from left inferior frontal to hippocampus, and from left precuneus to fusiform gyrus. Finally, with respect to the resting state, significant correlations were noted from left occipital temporal regions with hippocampus, from left supramarginal area with hippocampus, from left precuneus with hippocampus, from left inferior frontal with hippocampus, and from left precuneus with fusiform gyrus. The patterns of correlation also differed across groups suggesting different neurodevelopmental patterns and/or differing neural systems that may be involved in various learning problems.

### 3.4. Summary

**Figure 2:**
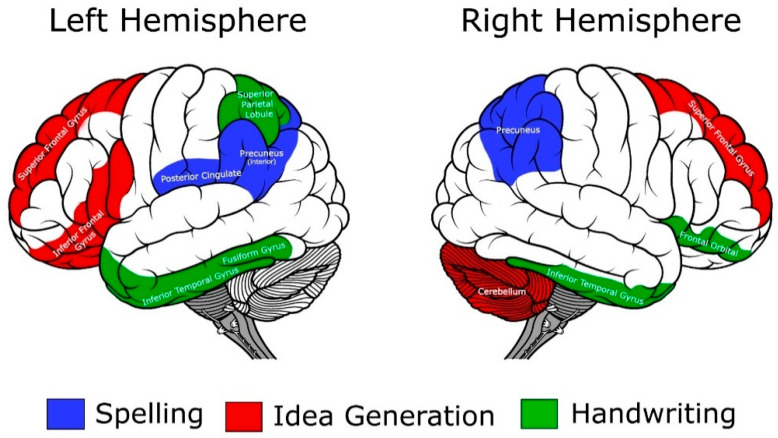
A composite of approximate brain regions associated with writing tasks.

Our scoping review identified 13 studies that have examined the neural underpinnings of writing in English speaking children and adolescents, ages 5–15. Results from these 13 studies revealed that good versus poor writers manifested differential brain activation patterns during many tasks associated with written expression, with these patterns being different from not just typically developing writers but those with dyslexia as well. Good writers and spellers are more efficient than poor writers in their brain activation patterns [15]. Poor writers and spellers tend to use more and different brain areas than good writers to complete the same task, thus they are over-connected and present as more inefficient in the neural activity during the writing process. Specifically, good writers activate higher-order cognition regions (i.e., left and right superior frontal gyri) during idea generation and planning (precursors to text generation), and activate the superior frontal gyrus (i.e., higher-order executive function of translation) during spelling [17]. In contrast, poor writers overwork the frontal and cingulate regions during spelling tasks [15], the left middle frontal gyrus (associated with verbal working memory) during idea generation [17], and the left inferior frontal gyrus during planning [10]. They also activate an additional 13 regions across the brain during handwriting tasks [10], and writers with poorer handwriting show decreased neural efficiency within the inferior frontal gyrus region [9]. Relatedly, it also has been suspected that these overactivation patterns can lead to cognitive fatigue earlier in the writing process (i.e., they use more cognitive resources such as working memory and attention), and thus yield a poorer written product. To date, there are no neuroimaging findings that have been reported for text generation, but the studies on idea generation and planning provide potential insights into what we might see with text generation. A graphic summary of these findings for handwriting, spelling, and idea generation can be seen in Figure 2.

### 3.5. Related Research: Brain Changes following Educational Intervention for Writing Problems

An innovation in intervention science would be to employ neuroimaging procedures to detect differences in brain functioning following an educational intervention. While these types of intervention studies were not included in the scoping review criteria (i.e., observational studies), it is important to mention the studies that have used neuroimaging as an outcome measure. Two studies have been conducted where an educational intervention for writing was implemented and a neural signature was obtained in a pre–post design [9,19].

In sample of 42 students that were assigned to typically developing, dysgraphia, dyslexia, and OWL LD groupings, Richards, Berninger, et al. [19] used a computerized intervention for the written language components of subword letter writing, word spelling, and syntax composing. A pre–post design was employed using DTI and fMRI connectivity to detect brain-based changes in response to the instruction. Behavioral improvements in handwriting, oral sentence syntax construction, and spelling were evident across the groups with respect to their DTI measures, this study found no difference between diagnostic groups on DTI indices. In contrast, there was a time by diagnostic group effect with respect to changes in gray matter connectivity in the left lateral cingulate gyrus for the alphabet writing task and in the right inferior frontal gyrus for the fMRI spelling task, particularly for the dysgraphia group. Correlations between various brain regions also increased post-intervention for the alphabet handwriting task and the spelling task. Taken together, these findings suggest that targeted language-based instruction can impact changes in white and gray matter connectivity, particularly with respect to brain-based changes that can occur with respect to improvement in writing tasks.

In a separate aim of one of the studies described in the review, Richards, Abbott, et al. [9] also noted significant brain connectivity changes following a language-based intervention with children and adolescents in grades 4 through 9. On the three writing tasks conducted during post-intervention scanning, the pattern of correlations with the cingulo-opercular network changed, with novel correlations being noted. Specifically, for the alphabet writing task, new correlations emerged between phonological coding with the right cingulate gyrus; focused attention with right cingulum; and attention switching with right middle frontal gyrus. For the spelling task, new significant connections were noted between morphological processing with left superior frontal and left middle frontal gyri. While for the planning task, new correlations were noted for orthographic loop processing with right insula and right cingulum. In sum, these findings indicate that there is a positive brain response to targeted instruction and that the autoregulation of these connections may be changing to become more efficient in their operation.

## 4. Discussion

The primary purpose of this scoping review was to highlight the available literature examining the neurobiological underpinnings related to written expression in children. A limitation of our review was that gray literature was not able to be examined, which may have yielded additional research results. However, while the number of studies identified was relatively small, the available corpus of studies is highly informative with respect to an emerging body of literature that illustrates the complexity of written expression and what neurological underpinnings may be contributing to both good writers and poor writers. Further, the available body of work shows the importance of such investigations for the various components of written expression (i.e., handwriting, spelling, idea generation) and the different brain regions that can be involved with each of these components. Though results are far from seamless, characterizing the neuroimaging findings associated with these separate components could begin to paint a picture of writing functions, as well as the underlying neurodevelopment and associated brain function. From this scoping review, findings showed several major trends in understanding the neurological basis of writing.

First, by examining how activation in specific parts of the brain correlates with behavioral measures, we can better understand the components of writing. There are associations between the left fusiform gyrus (e.g., the visual word form area) and orthographic coding (i.e., handwriting) [8]. Additionally, spelling and written composition measures are significantly correlated with activity in the left posterior cingulate, left precuneus, and right precuneus regions [15]. Moreover, activation in the left superior parietal, right inferior orbital, right and left inferior temporal regions during sequential finger movement has been significantly correlated with handwriting, spelling, and composing [9]. These findings show some overlapping conclusions with the adult literature examining the neurological bases of written expression, though there are some key differences, e.g., [20,21,22,23]. 

Second, it is important to examine the type of imaging methodology employed across the studies. While white matter integrity was examined in several studies, in all 13 studies fMRI was used to examine one of the components of written expression. This is a judicious selection since all the writing components are dynamic in nature and related more to the functioning of underlying neural systems than specific brain structures, although the latter information provides clues as to what brain regions and associated neural networks may be fruitful for additional exploration. The use of fMRI has paved the way for use of other dynamic neuroimaging techniques, such as functional near infrared spectroscopy (fNIRS), where movement artifact can be addressed more readily and data can be collected in ecologically valid settings (e.g., classrooms) as opposed to the laboratory settings; or the use of innovative technology designed to minimize movement artifact during a writing task while in the scanner [24,25]; or scanning protocols to address extraneous factors that may impact the brain image obtained (e.g., scanner noise, use of quiet delays) [26,27].

Third, there are several additional observations of the fMRI studies ascertained from this search. The sample sizes for all the available studies were small, ranging from 19 to 51 participants, thus limiting the power to detect small but significant brain changes and differential activation patterns. The type of writing task used certainly was not standardized, with some tasks (e.g., sequential finger movements) being developed for a specific study, so replicability of findings may be challenging. Further, few of the studies thoroughly addressed the presence of comorbid conditions (i.e., ADHD, dyslexia), but other dysfunctions (e.g., depression, anxiety) that could have affected [6] the brain activation patterns, particularly for a performance task, have not yet been explored. Certainly, none of the studies addressed the ongoing questions and challenges related to development—a particularly important consideration when studying children and adolescents. The clear alignment with theoretical models of writing, such as the Not-So-Simple View of Writing [28] or the revised Hayes model [29], also was lacking. Additionally, it is important to note that the bulk of these studies comprised the work of two or three laboratories, and it will be important for other investigative teams to engage in the neurobiological basis of writing and writing disabilities. These study factors clearly will need to receive additional attention in future investigations.

Finally, this scoping review begs an important question in the study of underlying brain functions in children’s written language; namely, why have there not been more studies examining the neurological underpinnings in written expression in children? We would conjecture that this is not an oversight in the literature but, rather, a confluence of factors that have limited the development of this intersectional study of brain function and written expression in children. One major reason is that there remains limited overlap between education investigations and medical investigations. Educators typically are not trained in the biological sciences and, conversely, medical professionals are not trained in educational constructs, such as written expression. To further punctuate this point, most educational researchers do not have immediate access to neuroimaging tools (e.g., no access to a suitable scanner and scanning procedures) and the necessary expertise to assist in collecting, analyzing, and interpreting such data. This clearly calls for an increase in interdisciplinary teams whereby the neurological basis of educationally related questions can be examined. Another factor pertains to the lag in research examining written expression versus the focus on reading, and to some extent mathematics. It is clear that the attention devoted to writing research falls far short of the attention given to reading and math, and this is even reflected in the number of neuroimaging studies that have been conducted in reading and math versus writing. Lastly, with respect to written expression in children, suffice it to say that it is multidimensional in nature and one of the most complex functions we exert as human beings; consequently, its study, including its neurobiological underpinnings, remains challenging.

## 5. Conclusions

While the findings and interpretations from the studies discussed in this scoping review are beneficial, researchers have only begun to understand the neurobiological basis of written expression and written expression disorders. The studies that have been conducted thus far (illustrated compilation in Figure 2) have yielded significant results and demonstrate just how critical this type of research is to our understanding the underlying neural network of written expression in children and adolescents. To illustrate, many education systems are currently emphasizing the importance of writing-to-learn programs, making this the perfect time to expand upon what we know about the brain and cognitive processes to better inform educational programs for both good and poor writers. The pathways to better scientific integration and true translational science are beginning to emerge, and an open, ongoing dialogue of the ultimate benefits of a neuroscience–educational partnership should provide advances with respect to our understanding of targeted writing functions, learning to write over time, and the impact of evidence-based instruction on brain functions of which work is beginning to emerge [9,19]. With these encouraging changes in brain functioning secondary to educational interventions, there are a number of other related questions. Do these changes in the strength and pattern of connectivity persist beyond the immediate post-treatment effects? Does the amount and developmental timing of the intervention contribute to the degree of change in connectivity? What about the potential differential effects of different types of intervention? Would there be a different pattern or magnitude of response to treatment in the presence of writing disabilities plus other comorbidities? There is clearly a plethora of questions that these intervention studies raise, but the findings to date provide a strong foundation for future studies.

In addition to interdisciplinary collaborations, researchers need to continue to be innovative in their methods to allow for more complex writing tasks to occur during the selected scanning procedure while at the same time reducing motion artifact in the images. Finally, it is imperative to use multiple levels of measurement (e.g., behavioral, cognitive, neurobiological) with the same participants to fully understand the complexities inherent in written expression and its development. Drawing from the promising results of neuroimaging studies completed in the areas of reading, mathematics, and attention, as well as the child studies cited in this review on written expression, future researchers can benefit from using neuroimaging to construct a more complete model of written expression development, contribute to increased neuroscience–educational collaborations, and to inform the efficacy of potential evidence-based interventions.

## Figures and Tables

**Table 1:** A summary of neuroimaging studies examining brain structures and functions during various writing tasks in children.

Author(s)	Date	Population	Sample Size	Neuroimaging Methodology	Writing Task	Brain Regions Observed
Handwriting						
Richards et al. [6]	2011	5th graders	20	fMRI BOLD activation was measured during on (writing a newly taught pseudo-letter) and off (writing the real letter ‘a’) tasks	Writing pseudo-letter or real letter ‘a’ with a wooden stylus on a touch pad on chest at midline at a steady pace practiced outside the scanner	Poor writers showed significant activation in many more brain regions than good writers, as well as activation in unique regions. Good writers activated significantly more in the left fusiform gyrus than did poor writers.
Giminez et al. [7]	2014	5- and 6-year-olds	51 (5 left-handed students were later excluded)	fMRI activation was measured during 3 matching tasks; in one condition, stimuli were presented simultaneously, and after a small delay in the other condition	Phonological processing task; non-verbalize visual-symbol matching task; color-matching task	Poorer handwriting was associated with stronger activation of the right pars triangularis of the inferior frontal gyrus.
Richards et al. [8]	2015	Grades 4–9 (M = 12 yrs, 3 mos)	40 (15 F, 25 M); Typical = 9; Dysgraphia = 14; Dyslexia = 17)	Diffusion Tensor Imaging (DTI) scans and fMRI connectivity scales	Resting state, alphabet writing task, spelling writing task, planning task (planned in task, then completed composition outside of scanner)	On structural connectivity, Dysgraphia Group showed less white matter integrity in the bilateral anterior thalamic radiation, left cingulate, and forceps minor than either of the other 2 groups; and less integrity in the left cortical spinal tract when compared to Typicals. The Dysgraphia Group showed higher radial diffusivity in 7 white mater tracts in the left hemisphere than Dyslexia Group. For functional connectivity, the Dysgraphia Group showed greater functional connectivity during planning tasks in left-occipital temporal, left inferior frontal gyrus, and left precuneus, but not during the resting task. For Dysgraphia Group, DTI-fMRI functional connectivity correlations between the left supramarginal gyrus and the left cingulum the anterior cingulate gyrus, and between the left inferior frontal regions and the superior parietal lobule, superior frontal gyrus, and the precuneus cortex. No differences between Typical versus Dysgraphia Group on the resting condition or alphabet writing task, but did show greater functional connectivity than Typicals on planning for composing.
Richards, Abbott, Yagle, Peterson et al. [9]	2017	Grades 4-9	42	MRI, fMRI	Alphabet writing, spelling fill in letter task, planning task	Found significant fMRI connectivity with this network was uncovered in left cingulate gyrus during the fMRI alphabet writing task.
Richards, Abbott, and Berninger [10]	2016	Grades 4–9; dysgraphia and ADHD	13	fMRI	Resting state (mind wandering), alphabet writing, spelling fill in letter task, planning task	Presence of ADHD was significantly related to the degree of brain connectivity across 3 of 4 writing tasks. For mind wandering, significant correlations for the left occipital temporal with fusiform 2; left supramarginal with fusiform 2. For the transcription writing tasks, significant correlations between the left supramarginal region and Broca’s area for both alphabet writing and spelling tasks. For the planning task, no significant correlations were observed.
Richards, Berninger, Stock et al. [11]	2009	Fifth graders	20	fMRI BOLD activation was measured during on and off tasks	Finger tapping with and without sequencing	Left superior parietal, right inferior frontal orbital, right and left inferior temporal areas were found to be associated in good but not poor writers. BOLD activation in these areas was significantly correlated with handwriting, spelling, and composing.
Spelling						
Bitan et al. [12]	2007	9–15-year-olds	38	fMRI BOLD activation was measured during the task	Words spelled the same or not the same	During the spelling task, the left inferior/middle frontal gyri, superior/medial frontal gyri, superior/medial temporal gyri, thalamus, cuneus/calcarine sulcus, middle temporal gyrus, and the left inferior parietal lobule were active.
Booth et al. [13]	2007	9–15-year-olds	48 children completed the visual spelling task	fMRI BOLD activation was measured during the visual spelling task	Visual spelling	On the visual spelling task, developmental increases were found in the effective connectivity from calcarine to the superior temporal gyrus.
Berninger et al. [14]	2015	9–15-year-olds, grades 4–9	45 children (controls *n* = 9, dysgraphia *n* = 14, dyslexia *n* = 17, OWL LD *n* = 5)	T2-weighted fMRI BOLD activation was used during a word-specific spelling task, deciding whether a letter string pronounced as a real word with meaning is a correctly spelled real word. fMRI looked at 4 regions: left occipital temporal, left supramarginal gyrus, left precuneus, and left inferior frontal gyrus	Participants were asked to complete the word judgement task on which they had been trained prior to scanning	OWL LD had the least connectivity in left occipital temporal seed point and left supramarginal gyrus, OWL LD had the least connectivity. OWL LD were similar to controls in the left precuneus seed point, OWL LD were similar to controls. OWL LD had slightly more connectivity than controls in the left inferior frontal seed point, OWL LD had slightly more connectivity than controls. In all seed points, dysgraphia had more connectivity than controls, and dyslexia had the most connectivity.
Richards, Berninger, and Fayol [15]	2009	10–12-year-olds, mean age of 11 years, 4 months	19 (12 good writers, 7 poor writers)	Used structural MRI scans and fMRI scans during on and off tasks	Real-word spelling judgment and pseudoword spelling judgment	Significant correlations between WIAT II composition and brain activation in 3 areas: left posterior cingulated, left precuneus, and right precuneus. Positive correlations for good spellers’ brain activation and negative correlations for poor spellers’ activation
Richards, Berninger, Winn et al. [16]	2009	10- and 11-year-olds	30 (10 good spellers, 20 with spelling disability)	Structural MRI and fMRI scans measured during presentation of stimuli	0-back control and 2-back condition using colored pictures of sea creatures	Poor spellers activated more than the good spellers in the bilateral superior frontal, middle frontal, inferior frontal, anterior cingulum, left postcentral, and right superior frontal. Suggested differential activation of regions associated with working memory.
Idea Generation/Planning						
Berninger et al. [17]	2009	7 and 9 years old during intervention, 10 years old during the fMRI scanning	20 (12 good writers, 8 poor writers)	Structural MRI and fMRI scans measured during presentation of verbal task	On/off tasks. 1) Off task: rest. 2) On-task: prompt was “Think about what you learned this summer that you have never learned in school. In a little while, you will leave the scanner and write about this topic.”	Good writers had more activation in brain regions associated with access to concepts and higher order cognition (left and right superior frontal gyrus), language, and executive functions related to language (left inferior frontal), working memory (right middle frontal orbital gyrus), and coordination (right cerebellum); poor writers had more activation in the left hemisphere, associated with working memory (left middle frontal gyrus); suggests that poor writers are inefficient in engaging working memory during idea generation.
Wallis et al. [18]	2017	Students with transcription disabilities (dysgraphia and dyslexia); controls; grades 4–9	39	fMRI	Resting state (mind wandering), alphabet writing, spelling fill in letter task, planning task	For the dysgraphia group, fMRI connectivity for 3 of 4 tasks was significantly related to one or more of the composition outcomes created outside the scanner. Handwriting was the only exception. The significant correlations for spelling were observed from left precuneus with the fusiform gyrus and amygdala, and from left inferior frontal gyrus with Broca’s area and fusiform gyrus. For planning, the connectivity was observed with left precuneus with cingulate, from left inferior frontal to hippocampus, and from left precuneus to fusiform gyrus. For resting state, significant correlations were noted from left occipital temporal regions with hippocampus, from left supramarginal area with hippocampus, from left precuneus with hippocampus, from left inferior frontal with hippocampus, and from left precuneus with fusiform gyrus. The patterns of correlation also differed across groups.

## Data Availability

Not applicable.
